# Anticipating and treating dementia: lessons hidden in plain sight

**DOI:** 10.1038/s41746-020-00359-3

**Published:** 2020-11-20

**Authors:** Leia Wedlund, Joseph Kvedar, Wanda Layman

**Affiliations:** 1 Harvard Medical School, Boston, MA USA; 2 Partners HealthCare, Boston, MA USA; 3 Nature Research, New York, NY USA

**Keywords:** Predictive markers, Diagnostic markers, Prognostic markers

Observation of patterns has long been a cornerstone of medicine and scientific discovery. Since Gregor Mendel first recorded pea plant inheritance patterns in the 1800s, the scientific method has not changed. Two hundred years later we continue to follow the same recipe: observe, hypothesize, test. What has changed since Mendel’s time is our access to data. Given millions of electronic health records and the analytical power of artificial intelligence, we are now able to detect patterns far more subtle than the inheritance of pea pod color.

The recent work of Park et al.^1^ (“Machine learning prediction of incidence of Alzheimer’s disease (AD) using large-scale administrative health data”) provides an exciting example of how machine learning can identify patterns that are nonobvious to human observation. Recognition of these new associations in turn leads to opportunities for earlier disease recognition and research on underappreciated risk factors/treatments^2^.

Park and Cho used health data from the Korean National Health Insurance Service database to train three different machine learning algorithms that predict the incidence of AD in adults over 65. They tested the predictive power of these algorithms over 1, 2, 3, and 4 subsequent years, comparing their predictions with recorded clinical diagnoses of AD confirmed by both ICD-10 code and dementia medication prescription. Their highest-performing algorithm was able to predict development of AD 1 year out with 71.3% accuracy.

What is remarkable about this work is that a relatively high level of predictive accuracy was achieved using commonly tested/recorded laboratories, medications, and medical history data. The algorithms developed did not rely on all patients undergoing expensive magnetic resonance imaging (MRI) or computed tomography head imaging, invasive cerebrospinal fluid (CSF) testing, or specialized memory and executive function tests such as a Mini Mental-State Examination (MMSE), which is typically only administered in scenarios where a clinician already suspects cognitive impairment.

By examining area under the curve (AUC), we can compare the overall diagnostic performance of Park and Cho’s algorithm with tests that require much more specialized data inputs (such as MMSE scores and cerebrospinal fluid studies). AUC is the probability that a person with the disease of interest will score higher on a given test than someone who is healthy^3^. Park and Cho’s algorithm had an AUC similar to that of models that predict progression from Mild Cognitive Impairment to Alzheimer’s disease using much more-specific neurologic studies such as MRI findings, MMSE score, and CSF biomarkers^4,5^. This illustrates that great predictive accuracy can be achieved using a simple national health database, and suggests that analysis of the existing health record could provide clinically useful screening for AD risk prior to any neuro-specific testing.

In addition, Park and Cho’s use of logistic regression, a modeling technique that describes the relative contribution of independent variables to overall risk of disease, allowed them to identify that health variables carried the greatest predictive weight, thus revealing nonobvious risk factors and suggesting possible avenues of future prevention, treatment, and research. This analysis suggested that anemia and proteinuria (the abnormal finding of having protein in the urine) are significant risk factors for development of Alzheimer’s disease, and showed a negative association between AD and tolfenamic acid (a non-steroidal anti-inflammatory) as well as nicametate citrate (a vasodilator). These findings support the importance of ongoing dementia research focused on anemia^3^ and the disease-modifying effects of tolfenamic acid^6^, whereas the new recognition of nicametate citrate as a risk-modifying agent creates entirely new opportunities for study.

With this work, Park and Cho have taken a first step towards development of a widely applicable Alzheimer’s risk-stratification tool. But perhaps even more importantly, they have completed the first step of that classic recipe: observe, hypothesize, test. They demonstrate the value of machine learning, not as a black box, but as a tool to focus our pathophysiologic research.
